# The role of chemoradiotherapy and immunotherapy in stage III NSCLC

**DOI:** 10.3389/pore.2024.1611716

**Published:** 2024-04-19

**Authors:** Zsuzsanna Orosz, Árpád Kovács

**Affiliations:** ^1^ Department of Pulmonology, Faculty of Medicine, University of Debrecen, Debrecen, Hungary; ^2^ Department of Oncoradiology, Faculty of Medicine, University of Debrecen, Debrecen, Hungary

**Keywords:** locally advanced, NSCLC, chemoradiotherapy, immunotherapy, durvalumab

## Abstract

Locally advanced non-small lung cancer encompasses a diverse range of tumors. In the last few years, the treatment of stage III unresectable non-small lung cancer has evolved significantly. The PACIFIC trial opened a new therapeutic era in the treatment of locally advanced NSCLC, establishing durvalumab consolidation therapy as the new standard of care worldwide. A careful evaluation of this type of lung cancer and a discussion of the management of these patients within a multidisciplinary team represents a crucial step in defining the best treatment strategy for each patient. For unresectable stage III NSCLC, definitive concurrent chemoradiotherapy (CCRT) was historically recommended as a treatment with a 5-year survival rate ranging from 20% to 30%. The PACIFIC study conducted in 2017 compared the use of chemoradiotherapy and maintenance therapy with the anti-PD-L1 monoclonal antibody durvalumab to a placebo in patients with locally advanced NSCLC who had not experienced disease progression. The study was prospective, randomized, and phase III. The administration of this medication in patients with locally advanced non-small cell lung cancer (NSCLC) has demonstrated a notable improvement in overall survival. Multiple clinical trials are currently exploring various immune checkpoint inhibition regimens to enhance the treatment efficacy in patients with stage III cancer. Our goal is to offer an up-to-date summary of the planned clinical trials for treatment options, focusing on the significant obstacles and prospects in the post-PACIFIC era.

## Introduction

Many diseases became more common as a result of the altered smoking habits that emerged in the first half of the 20th century and the air pollution that was seen to coincide with industrial development. Lung cancer is currently the most common cause of cancer-related mortality in developed nations [1]. The 5-year survival rates are 15%–40% in stage IIIA and 5%–10% in stage IIIB lung cancer, respectively [2]. Three subgroups exist for locally advanced stage III non-small-cell lung cancer (NSCLC) according to the 8th edition of the TNM classification: stage IIIA, IIIB, and IIIC tumors [2, 3]. However, in clinical practice, we can also identify stage lung cancer that is resectable, potentially resectable, and unresectable [4]. Lung cancer requires meticulous examination and staging [3]. The imaging and diagnostic methods include bronchoscopy, endobronchial ultrasound (EBUS), cryobiopsy, liquid biopsy, computed tomography (CT), FDG positron emission tomography (PET), magnetic resonance imaging (MRI), and in some cases it is necessary to perform video-assisted thoracoscopic surgery (VATS) [5]. After cytological or histological confirmation of non-small cell lung cancer, especially of adenocarcinomas, it is essential to determine the status of EGFR, ALK, and PDL1 is essential [5]. Therapy of stage III non-small cell lung cancer (NSCLC) needs a multimodal approach that involves chemotherapy, radiotherapy, surgical treatment, and in special cases targeted therapy and immunotherapy [2, 3].

Tumor location, volume, histology, immunohistochemical and molecular pathological features, patient age, performance level, and comorbidities are some of the variables that affect the treatment plan [1–3].

Before 2018, the standard of care therapy of locally advanced non-small cell lung cancer was definitive chemoradiotherapy, that contains platinum-based doublet chemotherapy regimen combined with either concurrent or sequential radiation therapy (RT) [6, 7]. The progression-free survival (PFS) was 8 months, and the 5-year survival was less than 20% in these patient population [8, 9].

A phase III clinical trial conducted in 2002 by Albain et al. showed that concomitant cisplatin, etoposide chemotherapy, and thoracic radiation improved the median overall survival (OS) to 15 months, with 3- and 5-year survival rates of 17% and 15%, respectively [10].

Subsequently, Govindan et al. reported that combination chemoradiotherapy using pemetrexed and carboplatin demonstrated fewer adverse effects while still demonstrating the same efficacy as cisplatin and etoposide [11].

## Chemoradiotherapy and immunotherapy for stage III unresectable NSCLC

The introduction of immune checkpoint inhibitors (ICI) opened a new era in the treatment of lung cancer 8 years ago [8, 12]. Previous studies showed the efficacy of immunotherapy after definitive chemoradiotherapy for stage III unresectable NSCLC. CRT is administered to reduce local recurrence and prevent the onset of distant metastases and is administered during or after the completion of chemoradiotherapy [12, 13].

The efficacy of durvalumab as maintenance therapy and the completion of concurrent chemoradiotherapy in locally advanced non-small cell lung cancer was assessed in 2017 by the phase III PACIFIC trial [12]. In the PACIFIC study, durvalumab (a human IgG1 monoclonal antibody against PD-L1) was added to concurrent chemoradiotherapy for stage III non-small cell lung cancer after a year of maintenance. 713 patients with stage III (TNM 7th edition) who had not received prior anticancer treatment were enrolled in the PACIFIC trial and were randomized to receive durvalumab or placebo beginning 1–42 days after combination chemoradiotherapy [5]. Concurrent radiotherapy (54–66 Gy) and platinum doublet chemotherapy (cisplatin or carboplatin plus paclitaxel, docetaxel, vinblastine, vinorelbine, etoposide, or pemetrexed) were administered to the patients [12]. Patients who did not progress after chemoradiotherapy were randomly assigned to receive durvalumab or placebo [12, 14]. Durvalumab was administered intravenously every 2 weeks at a dose of 10 mg/kg [12]. Durvalumab maintenance therapy for a year resulted in increases in overall survival (47.5 vs. 42.9 months) and progression-free survival (PFS; 11.2 vs. 10.9 months), with about one-third of patients continuing to live without distant metastases or local recurrence. Better 5-year OS (42.9% vs. 33.4%) and median OS (47.5 months vs. 29.1 months) were reported in the updated analysis for 2022 [13, 15].

In addition, patients treated with consolidation durvalumab had a longer median time to death or distant metastases (28.3 months against 16.2 months) and a lower incidence of brain metastases (6.3% vs. 11.8%) compared to placebo [13, 14, 16]. The efficacy of durvalumab in combination with concurrent or sequential radiation therapy in patients engaged in an early access program is being investigated in real-world data by an international retrospective trial known as PACIFIC-R [17]. Compared to PD-L1 negative patients, PD-L1 positive patients had a longer PFS (22.4 vs. 15.6 months) [13]. concurrent chemoradiotherapy with durvalumab is the current standard of care [5, 12, 18].

## The optimal sequence of chemoradiotherapy and immunotherapy

Sequential radiochemotherapy is less effective than concurrent platinum-based chemoradiotherapy (CRT) [6, 10, 19, 20]. However, the patient’s age, overall health, comorbidities, financial situation, and logistical challenges all affect the availability of competing radiation therapies [20].

The timing of immunotherapy is under investigation. Sequential immunotherapy is preferable because it has fewer side effects, can enhance treatment outcomes, and prevents resistance to immunotherapy through the immune system’s interaction with radiation [12]. According to the PACIFIC study’s subgroup analysis, patients who started their ICI earlier, in 30 days had a greater OS rate at 30 months compared to patients who received durvalumab after 1 months following chemoradiotherapy (90% vs. 44%) [7, 10].

In the LUN 14-179 [21], GEMSTONE-301 [22, 23], PACIFIC-6 [24], DETERRED [25, 26], and KEYNOTE 799 [26, 27] trials, immunotherapy was administered as a maintenance therapy after concurrent chemoradiotherapy.

In phase II DETERRED trial, chemoradiotherapy with concurrent and consolidative atezolizumab led to an efficacy similar to consolidative durvalumab in the PACIFIC trial [25, 26]. The patients received in part 1 chemoradiotherapy and consolidation of atezolizumab treatment, in part 2 they took concurrent maintenance atezolizumab. An updated analysis of this trial showed that the median progression-free survival for concurrent vs sequential atezolizumab was 15.1 vs. 18.9 months [25, 28].

The phase III KEYNOTE 799 study is similar to the PACIFIC trial. The KEYNOTE 799 trial evaluated the safety and efficacy of pembrolizumab and concurrent chemoradiotherapy in stage III non-small cell lung cancer. KEYNOTE 799 demonstrated an increase in ORR and an accelerated effect in establishing the antitumor immune response [27].

Phase III GEMSTONE 301 compared the efficacy of sugemalimab after chemoradiotherapy in patients with stage III unresectable driver mutation negative stage III NSCLC in China [22, 23]. PFS was significantly longer with sugemalimab than with placebo (median 10.5 vs. 6.2 months), but OS in the sugemalimab and placebo groups was inconclusive [29].

Phase II trial LUN 14–179 evaluates the efficacy and safety of pembrolizumab as consolidation therapy after concurrent chemoradiotherapy [21]. Consolidation with pembrolizumab after chemoradiotherapy prolonged PFS and OS compared to chemoradiotherapy (CRT) alone and did not increase the rates of grade 3–5 pneumonitis [21].

The single-arm phase II NICOLAS trial demonstrated the safety of nivolumab combined with radiation therapy in stage IIIA and IIIB disease. Significant overall survival differences were observed between patients with stage IIIA vs IIIB disease (2-year OS 81% vs. 56%) [30].

## Novel agents

The ongoing phase III PACIFIC-8 study investigates the efficacy and safety of domvanalimab and durvalumab compared to durvalumab. The phase III PACIFIC-9 trial compares durvalumab + chemotherapy treatment with the combination of oleclumab (CD73 inhibitor) or added monalizumab (NKG2 inhibitor). The SKYSCRAPER-03 study investigates consolidation therapy with atezolizumab and the TIGIT inhibitor tiragolumab after chemoradiotherapy. CHORUS is a phase III study that compares canakinumab combined with chemoradiotherapy and durvalumab. The KEYVIBE-006 study evaluated pembrolizumab/vibostolimab (TIGIT inhibitor) in combination with concurrent chemoradiotherapy followed by pembrolizumab/vibostolimab versus cCRT followed by durvalumab. The KEYLYNK-012 study assesses pembrolizumab in combination with concurrent chemoradiotherapy followed by pembrolizumab with olaparib placebo or olaparib compared to concurrent chemoradiotherapy followed by durvalumab.

## Next step: immunoradiotherapy, treatment without chemotherapy

In an attempt to avoid overtreating patients while dealing with the side effects of chemotherapy, a novel strategy known as radiation therapy with immunotherapy has been developed. The ongoing SPRINT study aims to evaluate the efficacy of a shortened 4-week radiation therapy course for high-grade PD-L1 patients with locally advanced non-small cell lung disease [31, 32]. The DUART trial, a phase II single-arm study, was finished to evaluate durvalumab’s clinical efficacy in patients with non-small cell lung cancer (NSCLC) that is incurable and not amenable to treatment [31]. In the finished PARTICLE-D trial, durvalumab, the study drug, and proton beam therapy were combined [32]. The active phase I NRG-LU004 trial examines durvalumab in combination with conventionally fractionated radiation therapy or accelerated hypofractionated radiation therapy (ACRT) in patients with locally advanced non-small cell lung cancer. It also investigate the safety of combining durvalumab with conventional radiation therapy in addition to monalizumab or oleclumab [33]. CHECKMATE 73L is a trial that is currently in progress. The main objective of the study is to examine the effectiveness of nivolumab plus concurrent chemoradiotherapy (CCRT) followed by nivolumab plus ipilimumab against CCRT followed by durvalumab in patients with untreated stage III unresectable non-small cell lung cancer [34].

## Driver mutations and advanced non-small lung cancer treatment

The therapy of non-small cell lung cancer with driver mutations in stage III disease raises many questions [35]. Targeted therapies especially tyrosine kinase inhibitors are evaluated in unresectable stage III NSCLC with actionable genomic alterations.

Unfortunately, there is no optimal treatment strategy for patients with driver mutation in locally advanced NSCLC. The PACIFIC trial enrolled patients with the EGFR mutation. Antonia et al. presented their exploratory subgroup analysis, showing that PFS and OS with durvalumab were similar to placebo in patients with EGFR mutation [15, 36, 37]. Hellyer et al. found that patients with LA- NSCLC mutations of the human epidermal growth factor receptor 2 (HER2) or EGFR had shorter PFS than those with wild-type genes (7.5 months vs. not reached) [35, 38]. We must use these data with caution due to the small number and characteristics of the patients (male, smokers, squamous cell carcinoma). The safety profile was the same in the durvalumab arm as in the overall population [15, 30]. The DETERRED trial examined individuals with specific oncogene mutations that can be targeted, which resulted in a poorer progression-free survival (PFS) [25]. The LAURA (phase III) trial enrolled patients with locally advanced, unresectable epidermal growth factor receptor mutation-positive stage III NSCLC. Patients received at least two cycles of concurrent/sequential platinum-based cCRT and were randomly assigned 2:1–80 mg or placebo once a day [39]. Currently, the effectiveness of osimertinib treatment is under investigation. The latest consensus of ESMO guideline does not recommend using ICI consolidation therapy after curative intention chemoradiotherapy in EGFR-positive NSCLC [3, 5, 40]. Patients with a KRAS mutation profit from durvalumab maintenance therapy [38, 41, 42].

## Toxicity and safety

Immunotherapy can affect multiple organ systems, 50% of patients treated with ICI are estimated to experience some form of irAE. AE of any grade of those who receive ICI treatment is fatigue, gastrointestinal (colitis, diarrhoea, abdominal pain, hepatitis), endocrine (alteration of thyroid function, hypocalcemia), myocarditis, renal, peripheral neuropathy, and dermatological side effects (i.e., rash). Respiratory complications, pneumonitis, and respiratory failure are the most common cause of immune-related deaths [12–14, 43–47].

Treatment-related pneumonitis with immune checkpoint inhibitors is a challenging side effect. The spectrum of symptoms moves on a wide range from the asymptomatic case to acute respiratory failure [48]. The diagnosis of ICI pneumonitis confirms the symptoms (developing dry cough, dyspnea, and other respiratory symptoms), laboratory test (inflammatory markers, i.e., neutrophil lymphocyte ratio), lung function test, microbial culture, the presence of new infiltrates on chest imaging without new infections [14, 43, 49]. Chest CT should be combined with other diagnostic tools. However, differential diagnosis is complicated and it is difficult to distinguish between radiotherapy-related pneumonitis, new-onset interstitial lung disease, tumour progression, or pulmonary infection (i.e., Covid infection) [48, 49].

Treatment-related adverse events of grade 3–4 were 29.9% in the PACIFIC trial. The most common AE was pneumonitis, with an incidence of 4.4%, and 15.4% of these patients discontinued durvalumab because of AEs [13, 14, 50].

With respect to adverse events, pneumonitis was reported to be more severe in patients who received durvalumab. However, grade 3 or 4 pneumonitis was similar in both groups: 1.9% in the durvalumab arm and 1.7% in the control group. Furthermore, radiation-related pneumonitis contributed to the discontinuation of durvalumab in 1.3% of patients, as in the placebo arm [12–15].

The novel combinations of immunochemoradiotherapy show similar data compared to those of the PACIFIC trial. In the GEMSTONE-301 trial, grade 3 or 4 pneumonitis occurred in 3% of patients in the sugemalimab group compared to 6% in the placebo group [22]. The grade 2 or higher pneumonitis rate was around 10% in the DETERRED trial [25].

Treatment of immune-related pneumonitis depends on the severity of the disease [51]. Grade 1 pneumonitis does not need special treatment. Grade 2 pneumonitis requires ICI withdrawal therapy and administration of corticosteroid treatment administration. Grade 3 and 4 pneumonitis requires discontinuation of immunotherapy, corticosteroid treatment, hospitalization, empiric broad-spectrum antibiotic therapy, oxygen administration, and mechanical ventilation in severe cases. Guidelines recommend other immunosuppressive agents: TNFα inhibitor - infliximab, intravenous immunoglobulins, and mycophenolat mofetil [51].

## Progression during and after ICI consolidation therapy

Regular chest CT or PET CT controls the efficacy of disease treatment and follow-up. PET-CT scan is recommended to evaluate tumor metabolic activity if it suggests progression of the disease [12]. Changes in F-18 fluorodeoxyglucose uptake occur commonly between 8 and 12 weeks after radiation therapy on PET CT [52]. Furthermore, several conditions (such as atelectasis, consolidation, infection, granulomatous pulmonary disease, and radiation fibrosis) are challenging to differentiate from neoplasm because areas previously treated with radiation therapy can remain avid 18F-FDG for up to 2 years.

More than half patients with stage III disease will progress within 2 years of the start of treatment. Updated data from the PACIFIC trial showed that 49.0% of patients completed 12 months of durvalumab therapy, and 31.3% discontinued due to disease progression [13]. In the PACIFIC trial, 7.1% of the patients in the ICI arm received durvalumab retreatment and completed the first year of consolidation immunotherapy, and the median time to second progression was 48 months [13, 24].

We must distinguish between oligo- and systemic progression. If primary resistance is confirmed (progression observed during durvalumab treatment), therapeutic strategies depend on the type of progression. Oligometastasis is treatable (e.g., bone, brain) with surgery or stereotaxic ablation; a tight follow-up is recommended. In systemic progression, the rechallenge of immunotherapy is doubtful and participation in clinical trials, including a PDL-1 inhibitor, can be recommended. Delasos et al. in their retrospective examination investigated pembrolizumab treatment after chemoradiotherapy and maintenance immunotherapy (durvalumab) [53]. Patients with refractory or recurrent NSCLC require more attention, their survival is worse (median OS 10.6 months, median PFS 6.1 months) than patients with metastatic NSCLC diagnosed *de novo* and treated with chemotherapy (median OS 12.9 months) [53].

## Conclusion

Treatments for locally advanced, incurable NSCLC have been evolving quickly in the field of lung cancer therapy in recent years. When treating unresectable stage III non-small lung cancer, chemotherapy plus immunotherapy have demonstrated a synergistic effect on both local and distant tumor control. The current therapeutic recommendations for unresectable stage III NSCLC include chemotherapy and 1 year of ICI consolidation therapy; however, there are a number of unanswered problems and potential techniques. We are currently awaiting the results to establish the best time to administer chemotherapy, radiation, and ICI as well as the function of targeted therapy. One major clinical barrier to improving the prognosis of patients with advanced lung cancer is resistance to immune checkpoint inhibitors.

## Abbreviations

ICIs, Immune checkpoint inhibitors; RT, Radiotherapy; LA-NSCLC, locally advanced non-small cell lung cancer; NSCLC, Non-small cell lung cancer; LA, Locally Advanced; SoC, Standard of care; OS, Overall survival; ORR, Objective Response Rate; TME, tumor microenvironment; PD-1, Programmed cell death 1; PD-L1, Programmed cell death-ligand 1; CTLA-4, Cytotoxic T-lymphocyte associated antigen 4; PFS, Progression-free survival; CRT, Chemoradiotherapy; AEs, Adverse events; PR, Partial response.
